# Elderly patients in crisis: unveiling outcomes and management approaches in severe COVID-19 cases - a retrospective analysis from Brazil

**DOI:** 10.31744/einstein_journal/2025AO1428

**Published:** 2025-08-25

**Authors:** Mayara Cristina Debone, Ricardo Kenji Nawa, Daniel Lima da Rocha, Marilia Pilotto de Oliveira, Rosalina Aparecida Partezani Rodrigues, Luciana Kusumota

**Affiliations:** 1 Universidade de São Paulo Escola de Enfermagem de Ribeirão Preto Ribeirão Preto SP Brazil Escola de Enfermagem de Ribeirão Preto, Universidade de São Paulo, Ribeirão Preto, SP, Brazil.; 2 Faculdade Israelita de Ciências da Saúde Albert Einstein Hospital Israelita Albert Einstein São Paulo SP Brazil Faculdade Israelita de Ciências da Saúde Albert Einstein, Hospital Israelita Albert Einstein, São Paulo, SP, Brazil.; 3 Serviço de Atendimento Móvel de Urgência Prefeitura Municipal de Sertãozinho Sertãozinho SP Brazil Serviço de Atendimento Móvel de Urgência, Prefeitura Municipal de Sertãozinho, Sertãozinho, SP, Brazil.

**Keywords:** Aged, COVID-19, Acute kidney injury, Length of stay, Mortality, Hospital mortality, Critical care outcomes, Intensive care units

## Abstract

**Objective:**

To analyze the sociodemographic and clinical characteristics, comorbidities, therapeutic resources, and mortality rates of elderly patients with COVID-19 admitted to an intensive care unit.

**Methods:**

This retrospective cohort study included patients admitted to the intensive care unit of a large hospital in São Paulo, Brazil, from March 1, 2020, to March 31, 2021. Patients were categorized as adults (≥18 years) and elderly (≥60 years).

**Results:**

Of the 504 patients, 326 (64.7%) were elderly. Compared to the adults, elderly patients had significantly higher Simplified Acute Physiology Score 3 and Sequential Organ Failure Assessment scores on admission (p<0.001). They also had a greater prevalence of comorbidities, as reflected by a higher Charlson Comorbidity Index (p<0.001) and were frailer according to the Modified Frailty Index (p<0.001). The elderly group had a longer intensive care unit stay, with an average of 17±19 days and a hospital stay of 40±39 days, compared to the adults who had an average intensive care unit stay of 14±16 days and a hospital stay of 28±25 days. Hospital mortality was significantly higher among elderly patients (37.7%) than among adults (12.4%) (p<0.001), especially in those who developed acute kidney injury and required vasopressors and extracorporeal membrane oxygenation. The other independent risk factors for mortality were frailty, arrhythmias, and prior organ transplantation.

**Conclusion:**

Frail elderly intensive care unit patients with multiple comorbidities require more resources, have longer hospital stays, and face higher mortality rates. Early interventions targeting this population could improve survival.

## In Brief

A retrospective cohort study was conducted over one year in a private intensive care unit in Brazil among 504 patients, 326 of whom were elderly (=60 years). Patients were grouped as adults and elderly to compare clinical profiles, assistance resources, and mortality predictors, using multivariate analysis.

## Highlights

■ The elderly had higher severity, more comorbidities, and longer intensive care unit and hospital stays than adults.
■ They required more assistance resources and had higher hospital mortality.
■ Independent mortality predictors: age ≥60, frailty, arrhythmia, transplant, acute kidney injury, vasopressors and extracorporeal membrane oxygenation.

## Graphical abstract

**Figure:**
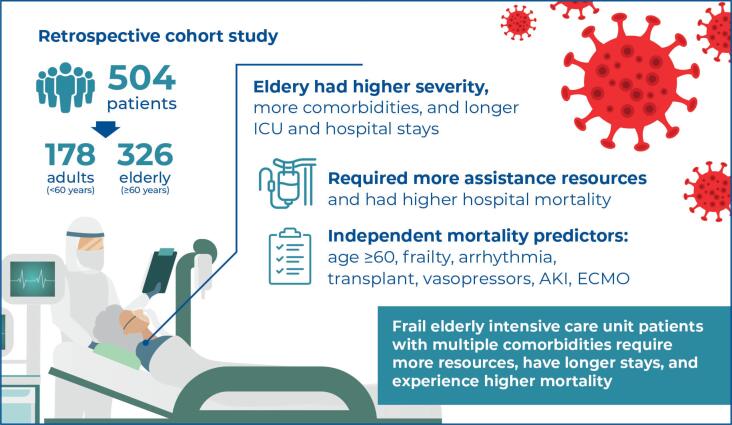

## INTRODUCTION

The coronavirus disease 2019 (COVID-19), caused by severe acute respiratory syndrome coronavirus 2 (SARS-CoV-2),^(1)^ was declared a pandemic by the World Health Organization on March 11, 2020.^(2)^ Over the past four years, in addition to infecting more than 775 million people, SARS-CoV-2 has caused approximately 7 million deaths globally,^(3)^ including over 700,000 in Brazil.^(4)^

Elderly individuals, in various countries, have been identified as the population segment with the highest mortality rate from COVID-19. In China, where the first cases of the disease were reported, an analysis of 72,000 cases registered by the Chinese Center for Disease Control and Prevention revealed a mortality rate of 2.3% in the general population. This rate was 8% among individuals aged 70-79 years and 14.8% among those over 80 years.^(5)^ In the United States, data from the first quarter of 2020 from the Centers for Disease Control and Prevention indicated a mortality rate of 10% to 27% in individuals aged 85 years and older. For those aged 65 to 84 years the rate varied from 3% to 11%, while it ranged from 1% to 3% among individuals aged 55 to 64 years. For patients aged from 20 to 54 years, the mortality rate was below 1%.^(6)^ In Italy, the *Istituto Superiore di Sanità* reported a mortality rate of 8.6% among those aged 60 to 69 years, 35.6% among those aged 70 to 79 years, and 52.3% in individuals aged 80 years and older, by March 2020.^(7)^

In Brazil, the distribution of deaths by age-group followed the trend observed in other countries. A study that analyzed COVID-19 deaths in Brazil during 2020 and 2021, categorizing over 631,000 deaths by age and sex, revealed that the highest mortality rates were in older age groups, ranging from 43.0 per 10,000 inhabitants aged 60 to 69 years to 149.0 per 10,000 inhabitants aged 80 to 89 years.^(8)^ This trend was already evident in the early months of the pandemic in Brazil. Between April and June 2020, the study showed that 62% of the deaths occurred in people aged 60 years or older, strongly associated with the presence of comorbidities such as hypertension (40%) and diabetes (31%).^(9)^

The clinical spectrum of COVID-19 ranges from asymptomatic or mild cases to severe conditions requiring hospitalization and intensive care unit (ICU) support.^(10)^ A Chinese study reported that severe symptoms, such as dyspnea, hypoxia, and pulmonary involvement occurred in approximately 14% of the cases, with 5% progressing to critical conditions such as respiratory failure, shock, or multiple organ failure.^(5)^ In a U.S. study that analyzed patients with severe COVID-19 symptoms, with an average age of 70 years, approximately 71% developed acute respiratory distress syndrome and required invasive mechanical ventilation. Additionally, 67% required vasopressors, 33% experienced cardiac issues, 20% developed acute kidney injury (AKI), and 15% experienced liver dysfunction.^(11)^

Comorbidities such as cardiovascular disease, diabetes, hypertension, chronic obstructive pulmonary disease, neoplasms, chronic kidney disease, obesity, and smoking are associated with manifestations of severe COVID-19.^(12)^ It is worth noting that these chronic diseases are common in the elderly population, and the accumulation of senescent changes in the immune system further increases their vulnerability to emerging infections, as seen in SARS-CoV-1, Middle East Respiratory Syndrome (MERS) Chikungunya (CHIKV), and, more recently, SARS-CoV-2.^(13)^ During the pandemic, which officially ended on May 5, 2023, and in the post-pandemic period, the epidemiological context of COVID-19 has changed.^(14)^ Although vaccination has significantly reduced mortality among elderly patients, it is important to recognize that this population remains at high risk for unfavorable outcomes, especially when associated with comorbidities, frailty, dependency in activities of daily living, and vulnerability, which are common in this phase of life.^(15)^ Thus, immunosenescence not only increases susceptibility to SARS-CoV-2 infection in the elderly but also limits the immune response to vaccines, potentially reducing their effectiveness compared to younger individuals.^(16)^

This study addresses a critical demand in clinical practice by providing specific insights into the characteristics and outcomes of elderly patients with COVID-19. These findings support nurses and multidisciplinary intensive care teams in identifying patients at high risk for poor outcomes, optimizing resource allocation, and planning targeted interventions. It highlights the role of nurses in coordinating care, managing resources, and improving strategies to ensure quality and safety in the care of critically ill elderly patients since hospital admission.

## OBJECTIVE

The study aimed to analyze and compare demographic and clinical characteristics, including comorbidities, as well as to describe the use of therapeutic resources and to identify mortality predictors in adult and elderly patients with SARS-CoV-2 admitted to an intensive care unit in Brazil.

## METHODS

### Study design and location

This was a retrospective cohort study conducted in compliance with the guidelines set forth in the Checklist for Reporting Results of Internet E-Surveys and the Strengthening the Reporting of Observational Studies in Epidemiology (STROBE) for cross-sectional studies.^(17)^ The study took place at a single center, a private quaternary care hospital in São Paulo (SP), which has 634 beds, including 37 in the adult medical-surgical ICU, during the research period. Local institutional review boards approved this study, as follows: *Hospital Israelita Albert Einstein* (HIAE), CAAE: 40178820.4.3001.0071; # 5.628.948. *Escola de Enfermagem de Ribeirão Preto da Universidade de São Paulo* (EERP/USP), CAAE: 40178820.4.0000.5393; 4.423.356.

Written informed consent from the participants was waived. During the COVID-19 pandemic, the hospital adapted an additional 44 stepdown unit care beds for intensive care, increasing the total to 81 beds. To minimize bias, these contingency beds were excluded from the study.

### Study participants

Patients aged ≥18 years admitted to the ICU at HIAE between March 1, 2020 and March 31, 2021 were included in the study if their COVID-19 diagnosis was confirmed by RT-PCR (Cobas^®^ SARS-CoV-2, Roche Molecular Systems, Branchburg, NJ, USA).^(18)^ Intensive care unit admissions followed institutional criteria and required at least one of the following conditions: acute respiratory failure requiring invasive mechanical ventilation (IMV), non-invasive ventilation (NIV), or high-flow nasal cannula (HFNC) with: FiO_2_ (fraction of inspired oxygen) >80%; or positive pressure with a delta >10cmH_2_O or EPAP (expiratory positive airway pressure) >10cmH_2_O to maintain SpO_2_ (peripheral oxygen saturation) >92%; or respiratory rate >24 breaths per minute during HFNC or NIV use; or PaCO_2_ (partial pressure of carbon dioxide) ≥50 mmHg with pH ≤7.35.

Other criteria included hemodynamic instability or shock, defined as: arterial hypotension (SBP, systolic blood pressure <90mmHg or MAP, mean arterial pressure <65mmHg); signs of organ or peripheral hypoperfusion (altered consciousness, oliguria, lactate ≥36mg/dL), with or without the use of vasopressors; sepsis, including septic shock, characterized by arterial hypotension, vasopressor requirement, or lactate ≥36mg/dL.

**Figure 1:**
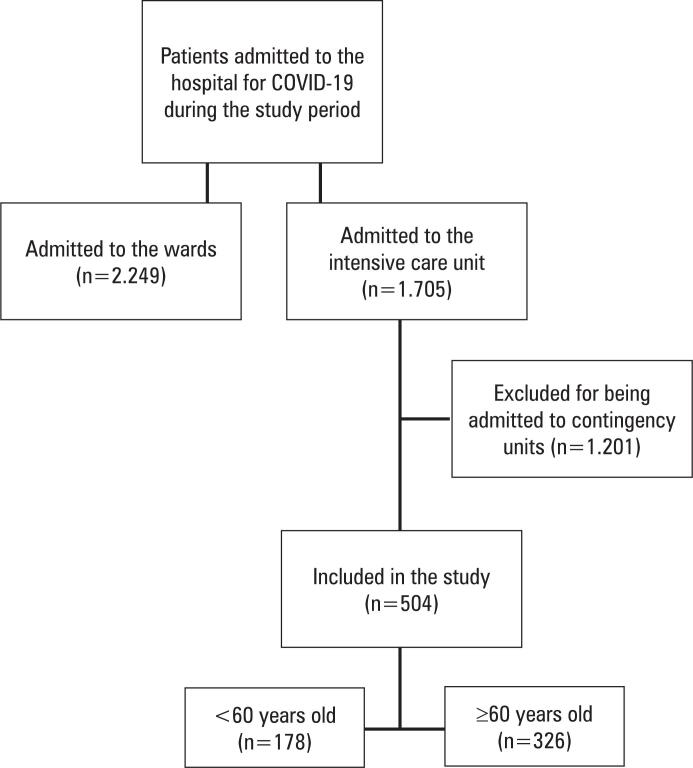
Eligibility and patient selection flowchart

The eligibility process, patient selection, and the representativeness of the cohort are outlined in figure 1.

### Data collection and variables

Data were extracted from the Epimed Monitor System (Epimed Solutions, Rio de Janeiro, Brazil)^(19)^ on April 14, 2021, by an independent research assistant. The collected variables included socio demographic data, comorbidities, Simplified Acute Physiology Score (SAPS 3),^(20)^ Sequential Organ Failure Assessment (SOFA),^(21)^ Charlson Comorbidity Index (CCI),^(22)^ Modified Frailty Index (mFI),^(23)^ and therapeutic resources utilized during hospitalization, such as vasopressors, non-invasive ventilation (NIV), extracorporeal membrane oxygenation (ECLS), among others. Outcome variables included ICU mortality within 28 days, overall ICU mortality, and hospital mortality.

### Statistical analysis

Descriptive analyses were performed to explore the characteristics of the data. Categorical variables were presented as absolute and relative frequencies, while continuous variables were summarized as means and standard deviations for data assumed to have a normal distribution, and as medians with interquartile ranges (IQR) for data assumed to have a non-normal distribution. Comparisons between quantitative variables were performed using the Student's *t*-test for normally distributed data, and the Mann-Whitney test for non-normally distributed data. Categorical variables were analyzed using the chi-square test or Fisher's exact test, as appropriate. Univariate models identified variables with p<0.10 for inclusion in multivariate models, and the likelihood ratio criterion was applied for stepwise variable selection. For the multivariate logistic regression model, variables with p<0.05 were retained. The models’ discrimination was evaluated using the area under the ROC curve (AUC), and calibration was assessed with the Hosmer-Lemeshow test. All analyses were performed using the SPSS software (IBM Corp. Released 2016. IBM SPSS Statistics for Windows, Version 24.0. Armonk, NY: IBM Corp.) with a 5% significance level.

## RESULTS

Between March 1, 2020 and March 31, 2021, a total of 504 patients aged ≥18 years, diagnosed with COVID-19 were admitted to the ICU of the studied hospital. The patients were divided into two groups: 178 adults ≥18 years (35.3%) and 326 elderly ≥60 years (64.7%). The median age of all the patients was 67 years (IQR 55–77], and 69.4% of the patients were male. Elderly patients demonstrated greater severity at admission, reflected by significantly higher SOFA and SAPS 3 scores than those observed in adults. The SAPS 3 score also indicated a substantially greater probability of death on admission among elderly patients, underscoring their higher clinical complexity and risk profile (Table 1).

**Table 1:** Socio-demographics, clinical characteristics, and comorbidities of adult and elderly patients treated for complications associated with SARS-CoV-2 in the intensive care unit

		All patients 504 (100%)	Patient Group		p value
			Adults 178 (35.3%)	Elderly 326 (64.7%)	
Age, years		67 [55-77]	51 [42-56]	74 [67-82]	-
Sex					
	Male	350 (69.4)	135 (75.8)	215 (66.0)	0.021*
	Female	154 (30.6)	43 (24.2)	111 (34.0)	
SAPS 3		51 [44-59]	44 [37-49]	55 [48-62]	<0.001†
Probability of death on admission from SAPS 3		18.9 [9.9-33.5]	9.9 [4.5-15.9]	25.7 [14.5-39.8]	<0.001†
SOFA		5 [3-8]	5 [2-6]	6 [4-8]	<0.001†
mFI		1 [0-2]	0 [0-1]	2 [1-3]	<0.001†
CCI		1 [0-2]	0 [0-1]	1 [0-2]	<0.001†
Comorbidities					
	Alcoholism	6 (1.2)	1 (0.6)	5 (1.5)	0.671‡
	Arrhythmia	19 (3.8)	2 (1.1)	17 (5.2)	0.021*
	COPD	41 (8.1)	1 (0.6)	40 (12.3)	<0.001*
	Dementia	37 (7.3)	1 (0.6)	36 (11.0)	<0.001*
	*Diabetes mellitus*	175 (34.7)	42 (23.6)	133 (40.8)	<0.001*
	Dialytic CKD	19 (3.8)	4 (2.2)	15 (4.6)	0.185*
	Non-dialytic CKD	34 (6.7)	6 (3.4)	28 (8.6)	0.026*
HF NYHA					0.001‡
	NYHA I	466 (92.5)	174 (97.8)	292 (89.6)	
	NYHA II or III	37 (7.3)	4 (2.2)	33 (10.1)	
	NYHA IV	1 (0.2)	0 (0.0)	1 (0.3)	
	Hypertension	282 (56.0)	66 (37.1)	216 (66.3)	<0.001*
MI, CAD or CABG		52 (10.3)	3 (1.7)	49 (15.0)	<0.001*
	Neoplasm	69 (13.7)	11 (6.2)	58 (17.8)	<0.001*
	Obesity	233 (30.3)	64 (36.0)	169 (52.3)	<0.001*
	Previous transplant	35 (6.9)	9 (5.1)	26 (8.0)	0.218*
	Psychiatric diagnosis	51 (10.1)	23 (12.9)	28 (8.6)	0.123*
	Smoking	23 (4.6)	4 (2.2)	19 (5.8)	0.066*
	Stroke	23 (4.6)	2 (1.1)	21 (6.4)	0.006*

Results expressed as median [IQR] or n (%).

Percentages may not sum to 100 due to rounding.

* χ^2^ test;

† Mann-Whitney test;

‡ Fisher's exact test.

CABG: coronary artery bypass grafting; CAD: coronary artery disease; CCI: Charlson Comorbidity Index; CKD: chronic kidney disease; COPD: chronic obstructive pulmonary disease; HF: heart failure; IQR: interquartile range; mFI: Modified Frailty Index; MI: myocardial infarction; NYHA: New York Heart Association; SAPS 3: Simplified Acute Physiology Score 3; SOFA: Sequential Organ Failure Assessment.

Elderly patients had higher comorbidities, with a median CCI of 1 [IQR 0–2] *versus* 0 [IQR 0–1] in adults (p<0.001), and greater frailty, with a median mFI of 2 [IQR 1–3] *versus* 0 [IQR 0–1] in adults (p<0.001) (Table 1). The main results related to hospitalization and clinical outcomes showed that elderly patients had longer ICU stays, with a median of 12 days [IQR 5–21], compared to 9 days [IQR 5–16] in adult patients. Similarly, hospital stays were longer for elderly patients, with a median of 29 days [IQR 16–48], compared to 20 days [IQR 12–33] in adults. Among the 145 hospital deaths, 123 (84.8%) occurred in elderly patients. Mortality was evaluated at three points: within 28 days in the ICU, during the ICU stay, and throughout the hospital stay. In all these time frames, the mortality rate was consistently higher in elderly patients compared to adults, with rates of 28.5% *versus* 7.9% within 28 days (p<0.001), 32.5% *versus* 10.7% in the ICU (p<0.001), and 37.7% *versus* 12.4% during the hospital stay (p<0.001). During hospitalization, a higher proportion of elderly patients received palliative care compared to adults (5.2% *versus* 0.6%) (p=0.007), and they also had higher incidence of AKI (48.5% *versus* 31.5%) (p<0.001) compared to the adults (Table 2). Due to sample size limitations, the association between delirium and sedation was evaluated without stratification by age. Among the 255 sedated patients, 51 (20%) developed delirium, whereas its incidence was 30 (12%) among the 249 non-sedated patients (p=0.002). Elderly patients received more blood component transfusions (133/326; 40.8%) compared to adults (48/178; 27.0%) (p=0.002). Similarly, renal replacement therapy (RRT) was utilized more frequently by elderly patients (117/326; 35.9%) compared to adults (43/178; 24.2%) (p=0.007). The use of cardiopulmonary resuscitation (CPR) was also higher among elderly patients (115/326; 35.3%) compared to adults (36/178; 20.2%) (p<0.001). While vasopressors were used more frequently by elderly patients (246/326; 75.5%) compared to adults (120/178; 67.4%), this difference did not reach statistical significance (p=0.053). Conversely, adult patients required more support with non-invasive ventilation (NIV) (116/178; 65.2%) compared to elderly patients (165/326; 50.6%) (p=0.002). A summary of therapeutic resources utilized by patients, varying according to age groups, can be seen in table 3.

**Table 2:** Hospitalization and outcome data of adult and elderly patients treated for complications associated with SARS-CoV-2 in the intensive care unit

		All patients 504 (100%)	Patient Group		p value
			Adults 178 (35.3%)	Elderly 326 (64.7%)	
Hospital stay		25 [14-44]	20 [12-33]	29 [16-48]	<0.001*
ICU stay		11 [5-19]	9 [5-16]	12 [5-21]	0.037*
ICU readmission		23 (4.6)	6 (3.4)	17 (5.2)	0.343†
Hospitalization data					
	AKI	214 (42.5)	56 (31.5)	158 (48.5)	<0.001†
	Delirium	81 (16.1)	23 (12.9)	58 (17.8)	0.155†
	Palliative care decision	18 (3.6)	1 (0.6)	17 (5.2)	0.007†
Mortality					
	Death within 28 days	107 (21.2)	14 (7.9)	93 (28.5)	<0.001†
	ICU death	125 (24.8)	19 (10.7)	106 (32.5)	<0.001†
	Hospital death	145 (28.8)	22 (12.4)	123 (37.7)	<0.001†

Results are expressed as median [interquartile range] or n (%).

Percentages may not sum to 100 due to rounding.

* Mann-Whitney test;

† χ^2^ test.

AKI: acute kidney injury; ICU: intensive care unit.

**Table 3:** Assistance resources and devices used by adult and elderly patients treated for complications associated with SARS-CoV-2 in the intensive care unit

	All patients 504 (100%)	Patient Group		p value
		Adults 178 (35.3%)	Elderly 326 (64.7%)	
CPR	151 (30.0)	36 (20.2)	115 (35.3)	<0.001
CVC	355 (70.4)	124 (69.7)	231 (70.9)	0.779
ECLS	28 (5.6)	14 (7.9)	14 (4.3)	0.094
HFNC	115 (22.8)	44 (24.7)	71 (21.8)	0.452
IMV	382 (75.8)	131 (73.6)	251 (77.0)	0.395
Indwelling urinary catheter	385 (76.4)	129 (72.5)	256 (78.5)	0.126
Invasive blood pressure monitoring	330 (65.5)	113 (63.5)	217 (66.6)	0.487
NIV	281 (55.8)	116 (65.2)	165 (50.6)	0.002
Parenteral nutrition	21 (4.2)	9 (5.1)	12 (3.7)	0.460
RRT	160 (31.7)	43 (24.2)	117 (35.9)	0.007
Sedation	255 (50.6)	89 (50.0)	166 (50.9)	0.843
Tracheostomy	79 (15.7)	22 (12.4)	57 (17.5)	0.130
Transfusion	181 (35.9)	48 (27.0)	133 (40.8)	0.002
Vasopressor	366 (72.6)	120 (67.4)	246 (75.5)	0.053

Results are expressed as n (%).

Percentages may not sum to 100 due to rounding.

Comparisons performed using χ^2^ tests.

CPR: cardiopulmonary resuscitation; CVC: central venous catheter; ECLS: extracorporeal life support; HFNC: high-flow nasal cannula; IMV: invasive mechanical ventilation; NIV: non-invasive ventilation; RRT: renal replacement therapy.

The univariate and multivariate regression analyses of risk factors associated with in-hospital mortality considered sociodemographic characteristics, pre-existing comorbidities, and the resources used during the ICU stay (Table 4). The multivariate analysis revealed that although variables such as SAPS 3 and SOFA were significant in the univariate analysis (p<0.001 for both), they lost their significance when analyzed alongside other variables in the multivariate model. In contrast, factors such as being an elderly patient (326/504; odds ratio [OR]= 1.8; 95% confidence interval [95%CI]: 1.424–2.297; p<0.001), having higher mFI scores (median 2 [IQR 1–3]; OR 1.4; 95%CI= 1.051–1.735; p=0.019), and developing AKI (214/504; OR 3.6; 95%CI= 2.000–6.337; p<0.001) were associated with higher risk of in-hospital mortality. Pre-existing conditions like arrhythmia (19/504; OR 3.8; 95%CI= 1.079–13.261; p = 0.038) and prior transplant (35/504; OR 5.1; 95%CI= 1.975–13.064; p<0.001) were also significant. The model also highlighted the role of ICU support resources in patient outcomes. Among the 28 patients who required ECLS, 14 were adults (7.9%) and 14 were elderly (4.3%) (p=0.094), with an OR of 4.4 (95%CI= 1.694–11.632; p=0.002) for in-hospital mortality. Similarly, vasopressors were used in 366 patients, with higher usage among elderly patients (246/326; 75.5%) compared to adults (120/178; 67.4%) (p=0.053) and showed a strong association with increased mortality risk (OR 5.2; 95%CI= 2.857–9.358; p<0.001).

**Table 4:** Univariate and multivariate logistic regression for the mortality outcome of patients treated for complications associated with SARS-CoV-2 in the intensive care unit

	Univariate analysis		p value	Multivariate analysis		p value
	OR	95% CI		OR	95% CI	
Elderly (≥60 years)	4.3	2.608-7.079	<0.001	1.8	1.424-2.297	<0.001
Male sex	1.5	0.958-2.294	0.077			
SAPS 3	1.1	1.038-1.063	<0.001			
SOFA	1.3	1.201-1.385	<0.001			
mFI*	1.7	1.400-1.957*	<0.001	1.4	1.051-1.735	0.019
Hospital stay	1.0	0.993-1.004	0.564			
ICU stay	1.0	1.014-1.036	<0.001			
AKI	6.6	4.298-10.233	<0.001	3.6	2.000-6.337	<0.001
Delirium	0.4	0.199-0.724	0.002			
Palliative care decision	9.5	3.067-29.339	<0.001			
CCI	1.4	1.220-1.535	<0.001			
Comorbidities						
Alcoholism	1.2	0.225-6.852	0.804			
Arrhythmia	2.3	0.918-5.807	0.075	3.8	1.079-13.261	0.038
COPD	2.3	1.209-4.412	0.011			
Dementia	1.8	0.889-3.513	0.104			
*Diabetes mellitus*	2.1	1.399-3.093	<0.001			
Dialytic CKD	2.3	0.918-5.807	0.075			
Non-dialytic CKD	1.6	0.771-3.258	0.210			
Heart failure NYHA: I. II. III or IV	2.1	1.093-4.182	0.026			
Hypertension	1.6	1.081-2.394	0.019			
MI, CAD or CABG	2.6	1.430-4.588	0.002			
Neoplasm	3.5	2.080-5.893	<0.001			
Obesity	0.9	0.611-1.137	0.669			
Previous transplant	6.3	2.985-13.192	<0.001	5.1	1.975-13.064	<0.001
Psychiatric diagnosis	0.7	0.326-1.315	0.234			
Smoking	1.6	0.690-3.856	0.265			
Stroke	1.3	0.555-3.231	0.516			
Assistance resources and devices						
CPR	4.8	3.146-7.237	<0.001			
CVC	4.7	2.705-8.322	<0.001			
ECLS	4.2	1.916-9.212	<0.001	4.4	1.694-11.632	0.002
HFNC	0.5	0.307-0.853	0.010			
IMV	8.0	3.776-16.814	<0.001			
Indwelling urinary catheter	7.7	3.631-16.181	<0.001			
Invasive blood pressure monitoring	6.9	3.882-12.227	<0.001			
NIV	0.2	0.155-0.355	<0.001	0.2	0.136-0.402	<0.001
Parenteral nutrition	2.3	0.973-5.645	0.058			
RRT	6.8	4.416-10.335	<0.001			
Sedation	1.8	1.247-2.739	0.002			
Tracheostomy	2.3	1.390-3.734	0.001			
Transfusion	4.1	2.699-6.079	<0.001			
Vasopressor	6.0	3.194-11.245	<0.001	5.2	2.857-9.358	<0.001

The multivariable model had an area under the receiver operating characteristic curve (95%CI) of 0.891 (0.864 – 0.918) and a Hosmer-Lemeshow χ² of 5.787 (p=0.671).

Odds ratios (OR) for SAPS 3, SOFA, hospital length of stay, ICU length of stay, and Charlson Comorbidity Index were calculated for a 1-unit increase.

* mFI: odds ratio calculated for each 0.1-point increase in score.

AKI: acute kidney injury; CABG: coronary artery bypass grafting; CAD: coronary artery disease; CCI: Charlson Comorbidity Index; CKD: chronic kidney disease; COPD: chronic obstructive pulmonary disease; CPR: cardiopulmonary resuscitation; CVC: central venous catheter; ECLS: extracorporeal life support; HFNC: high-flow nasal cannula; ICU: intensive care unit; IMV: invasive mechanical ventilation; mFI: Modified Frailty Index; MI: myocardial infarction; NIV: non-invasive ventilation; NYHA: New York Heart Association; RRT: renal replacement therapy; SAPS 3: Simplified Acute Physiology Score 3; SOFA: Sequential Organ Failure Assessment; 95% CI: 95% confidence interval.

**Figure 2:**
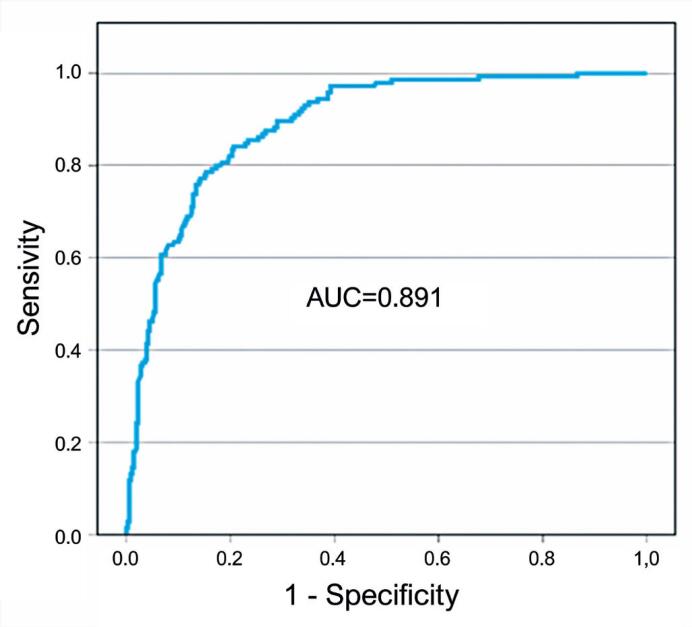
ROC curve for multivariate analysis predicting hospital mortality in COVID-19 ICU patients

Conversely, the use of NIV was associated with a lower risk of death. Among the 281 patients who required NIV support, the OR was 0.2 (95%CI= 0.136–0.402; p<0.001), with elderly patients showing less frequent use compared to adults (165/326 *versus* 116/178, respectively). The multivariate analysis revealed an AUC of 0.891, indicating that the model was effective in classifying hospital deaths (Figure 2). Additionally, the Hosmer-Lemeshow test produced a χ^2^ statistic of 5.787 with 8 degrees of freedom and a p-value of 0.671. Given this p-value, there was insufficient evidence to reject the null hypothesis, suggesting that the model was well-calibrated and reinforced its robustness in predicting in-hospital mortality.

AUC: area under the curve.

## DISCUSSION

This single-center retrospective cohort study analyzed data from 504 patients admitted to the ICU for COVID-19, 64.7% of whom were elderly. The higher incidence and severity of COVID-19 among elderly patients may be attributed to immunosenescence and congregate living environments that facilitate viral transmission.^(24)^

In this analysis, elderly patients had higher SAPS 3 and SOFA scores upon admission, reflecting greater disease severity and higher mortality rate (37.7%) compared to adults (12.4%). Previous studies support these findings, linking elevated SAPS 3 and SOFA scores to an increased likelihood of multi-organ failure and the need for intensive support, particularly in elderly patients.^(25)^ Advanced age has consistently been associated with unfavorable outcomes in COVID-19 patients.^(26,27)^ Even post-vaccination, over 80% of the global COVID-19 deaths occurred among elderly patients.^(28)^ Some authors have demonstrated that dysregulation in antibody production in the elderly increases susceptibility to severe COVID-19,^(29)^ while other authors have found that male sex, in addition to age, is associated with higher mortality rates, partly due to the presence of autoantibodies that neutralize type I interferons, thereby increasing disease severity.^(30)^

Data showed that 69.4% of the patients were men, who had significantly higher mortality rates than women. The increased severity of COVID-19 in men may be attributed to differences in immune function, such as reduced T-cell activation observed in elderly men.^(31)^ Additional studies confirm that male sex, advanced age, and comorbidities increase the risk of death from COVID-19.^(32,33)^ Elderly patients, in particular, exhibited a higher number of comorbidities, including arrhythmia, stroke, dementia, diabetes, COPD, hypertension, heart disease, neoplasms, and obesity, leading to higher CCI scores.^(33,34)^

Arrhythmias and previous transplants emerged as significant predictors of mortality in our cohort, with adjusted ORs of 3.8 and 5.1, respectively. Similar findings have been reported, with higher mortality rates observed in patients with cardiovascular diseases.^(34)^ Frailty, as measured by the mFI, was also a significant predictor of mortality, independent of age and comorbidities.^(35)^ Frailty, marked by reduced muscle function and homeostatic capacity, is strongly linked to poor outcomes, especially in elderly COVID-19 patients.^(35)^ The requirement for intensive medical intervention, such as vasopressors and ECLS, was another factor linked to higher mortality in this cohort. Patients needing vasopressors exhibited an increased likelihood of death, with their use associated with the development of AKI – a common complication in severe COVID-19 cases.^(36)^ In our cohort, AKI was also identified as a significant predictor of mortality.

Extracorporeal membrane oxygenation support, widely used during the pandemic to oxygenate patients with severe acute respiratory distress syndrome (ARDS), was associated with higher mortality rates, particularly among elderly patients, according to data from the Extracorporeal Life Support Organization.^(37)^ A meta-analysis also linked higher mortality rates to the use of ECLS in elderly patients, whereas younger and less severe patients had a better chance of recovery and survival with this support.^(38)^ It is important to note that the use of resources such as vasopressors, AKI requiring RRT, and ECLS should be understood as reflecting the severity of the patient's condition rather than serving as direct predictors of mortality. These interventions are utilized based on the critical state of the patient, which necessitates their use.

Patients who received NIV had lower mortality rates, likely due to less severe clinical conditions, that did not require intubation. At the center where this study was conducted, patients meeting intubation criteria—such as hypoxemic or hypercapnic respiratory failure, airway obstruction, impaired consciousness requiring airway protection, increased respiratory effort, and neuromuscular weakness—received appropriate support.^(39)^ Those who remained on NIV avoided the risks associated with invasive ventilation, including ventilator-induced lung injury, ventilator-associated pneumonia, hemodynamic instability, reduced respiratory muscle strength, the need for sedation, and prolonged immobility, avoiding these complications likely contributed to the more favorable outcomes observed.^(40)^

In the absence of a satisfactory response to therapeutic interventions, the decision to provide palliative care became crucial, particularly for elderly patients with advanced diseases. In the United States, 24.8% of intubated patients received palliative care consultations, with advanced age being a significant factor.^(41)^ This study underscores the importance of a multidisciplinary approach in managing critically ill COVID-19 patients, highlighting the paramount role of the nursing team in ensuring the humanization and dignity of patients through all stages of care.^(42)^

During the care of critically ill COVID-19 patients, the nursing team plays an important role in clinical monitoring, preventing complications, and supporting both patients and their relatives. Additionally, nursing contributes to maintaining a humanized approach, even in a highly technical environment, particularly in cases of prolonged hospital stays and increased mortality risks.^(42)^

Hospital mortality among critically ill COVID-19 patients was 28.8%, regardless of age. This figure aligns with the findings of a study that reviewed 73 ICUs worldwide and reported a combined mortality rate of 30%,^(43)^ as well as with a meta-analysis of 2,663 articles that found a combined mortality rate of 28.3%.^(44)^ This study enhances our understanding of risk factors in critically ill elderly COVID-19 patients and supports improved clinical management, personalized care, and public health policy development.

Data collection was conducted at a large hospital with advanced infrastructure using the Epimed Monitor System, minimizing bias through standardized data collection. This setup, combined with real-time data tracking, enabled detailed analysis of key clinical variables. However, the availability of extensive resources at this hospital, including advanced intensive care technologies and specialized staff, may limit the generalizability of the findings to other institutions with fewer resources, especially in regions with limited access to intensive care infrastructure. Future research should focus on multicenter studies and longitudinal follow-ups to validate these findings. Despite these limitations, this study offers valuable insights that may enhance clinical management and improve training programs of healthcare teams for future crises.

## CONCLUSION

This study demonstrated that elderly COVID-19 patients in the intensive care unit faced greater disease severity, a higher burden of comorbidities, longer hospital stays, and elevated mortality rates compared to adults. Key mortality predictors included advanced age, frailty, arrhythmias, prior organ transplants, acute kidney injury, vasopressor use, and reliance on extracorporeal membrane oxygenation. Timely interventions addressing these factors could improve survival outcomes. Nursing care remains essential for continuous monitoring, compassionate patient support, and guidance for relatives during these challenging times. These findings underscore the need for targeted strategies in managing elderly patients to improve outcomes in future health crises.
